# Epidemiology of sepsis in brazilian icus: a nationwide stratified sample

**DOI:** 10.1186/2197-425X-3-S1-A642

**Published:** 2015-10-01

**Authors:** FR Machado, AB Cavalcanti, F Bozza, DC Angus, EM Ferreira, F Carrara, J Lubarino, R Salomao, LC Pontes de Azevedo

**Affiliations:** 1 Latin American Sepsis Institute, Sao Paulo, Brazil; 2 Federal University of Sao Paulo, Sao Paulo, Brazil; 3 Brazilan Research in Intensive Care Network, Sao Paulo, Brazil; 4 Hospital do Coração Research Institute, Sao Paulo, Brazil; 5 AMIBnet, Sao Paulo, Brazil; 6 University of Pittsburgh, Pittsburgh, United States

## Introduction

Previous studies of the prevalence and fatality for sepsis in patients admitted to Brazilian intensive care units (ICU)^(^[1]^-^[3]^)^ were based on a small convenience sample, with most ICUs located in the wealthiest regions of the country. Thus, the current national sepsis prevalence, fatality rates and its associated risk factors are unknown.

## Objectives

To assess prevalence, fatality rates and prognostic factors of severe sepsis and septic shock, in a stratified random sample of Brazilian ICUs.

## Methods

A point prevalence study. The calculated sample size was 2,450 ICU beds to enroll 784 severe sepsis/septic shock patients. To generate the sampling frame, we created ten different strata, based on geoeconomic regions, considering hospitals located at state capitals or countryside and institutional profile (public or private), and randomly selected 13% of the ICUs in each stratum. All selected ICUs answered a websurvey regarding the availability of key clinical resources. We constructed a score based on the availability of 8 equal-weight items: ability to measure blood gases, lactate (both available in three hours); cultures (blood, urine and respiratory secretions), antibiotics (third-generation cephalosporins, carbapenems or piperacillin-tazobactam plus vancomycin or teicoplanin or linezolide), central venous catheters, crystalloids, norepinephrine and availability to measure central venous pressure. We considered as high availability those ICUs who always have all 8 items, intermediate availability those with 6-7 items and low availability those with 5 or less items. We included all patients with severe sepsis or septic shock hospitalized in the participants ICUs on one specific day (26/February/2014). We assessed demographic data, SAPS3 and compliance with sepsis treatment bundles and followed all patients until hospital discharge, truncated at 60 days.

## Results

On the day of study there were 794 (29.6%) patients with sepsis or septic shock among 2,705 patients already hospitalized or admitted in the 229 participant ICUs. The overall mortality rate was 55,7%. Mortality was 57.8% (67/116) in the South, 51.2% (208/407) in the Southeast, 70% (56/81) [f2] in Midwest, 58.3% (77/136) in Northeast and 57.4% (31/54) in the North region (P = 0.03). The mortality of public hospitals (56.0%; 253/452) was not different from the private ones (55.4%; 186/336). Factors independently associated with a higher mortality rate are in Table 1.

**Table 1:** Factors associated with mortality.

Variables	OR	95%CI	p value
SAPS score	1.04	1.03 - 1.05	< 0.001
Antibiotics compliance	0.67	0.46 - 0.97	0.04
6-h bundle compliance	0.48	0.31 - 0.75	0.001
Sepsis onset at ICU	1.64	1.12 - 2.42	0.01
Low availability of resources	1.79	1.04 - 3.09	0.04

## Conclusions

Sepsis prevalence and mortality in Brazilian ICU are very high and represent a significant burden in critical care. Mortality is heterogeneous throughout Brazilian regions and is related to patients' characteristics but also to resource availability and adequate processes of care.

## Grant Acknowledgment

FAPESP - 2011/20401-4.

## References

[1] Silva E, et al: Crit Care. 2004, 8 (4): R251-60. 10.1186/cc2892. 15312226 10.1186/cc2892 PMC522852

[2] Sales JAL, et al: Rev Bras Ter Intens. 2006, 18 (1): 9-17. 10.1590/S0103-507X2006000100003.

[3] Sogayar AM, et al: Pharmacoeconomics. 2008, 26 (5): 425-34. 10.2165/00019053-200826050-00006. 18429658 10.2165/00019053-200826050-00006

